# National survey of home injuries during the time of COVID-19: who is at risk?

**DOI:** 10.1186/s40621-020-00291-w

**Published:** 2020-11-11

**Authors:** Andrea C. Gielen, Grace Bachman, Oluwakemi Badaki-Makun, Renee M. Johnson, Eileen McDonald, Elise Omaki, Keshia M. Pollack Porter, Leticia Ryan, Wendy Shields

**Affiliations:** 1 Johns Hopkins Bloomberg School of Public Health, Department of Health, Behavior and Society, Johns Hopkins Center for Injury Research and Policy, 624 N. Broadway, Baltimore, MD 21205 USA; 2 Johns Hopkins Bloomberg School of Public Health, Department of Health, Behavior and Society, Baltimore, USA; 3 Johns Hopkins School of Medicine, Department of Pediatric Emergency Medicine, Baltimore, USA; 4 Johns Hopkins Bloomberg School of Public Health, Department of Mental Health, Johns Hopkins Center for Injury Research and Policy, Baltimore, USA; 5 Johns Hopkins Bloomberg School of Public Health, Department of Health Policy and Management, Johns Hopkins Center for Injury Research and Policy, Baltimore, USA

**Keywords:** Home injury, COVID-19, Pandemic

## Abstract

**Background:**

Prior to the COVID-19 pandemic, 44% of all reported injuries in U.S. households occurred in the home. Spending more time at home due to the pandemic may increase the number of home injuries.

**Methods:**

A nationally representative sample of 2011 U.S. adults were surveyed online between June 17 – June 29, 2020. Propensity score weighting and T-tests were used.

**Results:**

Twenty-eight percent (28%) of households reported a home injury or ingestion during the pandemic; 13% reported experiencing both. Injuries were most often due to falls (32%). Medication ingestions were reported by 6%; household product ingestions were reported by 4%. Relative to households that experienced no injuries or ingestions, those that reported either or both were more likely to: be in urban areas, have household incomes > $100,000, and have children living in them. Among households reporting more time spent at home, those with children were significantly more likely than those without to report an injury or ingestion.

**Conclusions:**

Results help target prevention messages while U.S. families are continuing to work and learn remotely. During this pandemic and future stay-at-home orders, there is a need for public health efforts to prevent home injuries and ingestions.

## Background

Injuries in and around the home, including ingestions, affect individuals across the lifespan (Mack et al. 2013; Gielen et al. 2015; McDonald et al. 2016). The National Health Interview Survey (NHIS) last reported data on injuries that occurred in U.S. households in 2007, finding that 44% of all reported medically attended injury episodes occurred in or around the home (Chen et al. 2009). The increased exposure to potentially hazardous home environments and activities caused by stay-at-home orders and closures of schools may be responsible for a new spate of home injuries in 2020. There is no near real time medical surveillance system for home injuries in the U.S., a gap that we address through the use of a national survey. This brief report describes the number and type of home injuries among a nationally representative sample of U.S. households surveyed between June 17 and 29, 2020, when states were at various stages of re-opening and had stay-at-home recommendations. To identify risk factors for these injuries, we compare household characteristics between those that reported injuries and/or ingestions to those that did not.

## Methods

We added home injury questions to an online survey of a representative sample of adults living in the U.S. conducted by the Harris Poll. Harris uses a large network of online panels with millions of unique respondents worldwide recruited through more than 100 different sources. All panelists complete a confirmed or double opt-in process, which is an approved proxy for informed consent that includes introductory language describing the survey. A total of 3211 individuals (of 3946) were qualified and selected to participate, with 2011 completing the survey. This survey was approved by the Johns Hopkins University Institutional Review Board.

Two survey items adapted from the NHIS (Chen et al. 2009) were used to assess injuries and ingestions (see Table 1). Affirmative responses were followed by questions about the external causes of the events (e.g., fall, burn, etc.) without reference to intentionality. Household level variables included: urbanicity (in an urban or city area; in a suburban area next to a city; or in a small town or rural area); Census region; annual household income; and number of adults and children living in the home. We also asked respondents about the impact of the pandemic on the amount of time household members spent at home (more time, less time, no change, varies by person). Households that reported experiencing *neither* an injury nor an ingestion were compared first to the households that reported experiencing *either* an injury *or* ingestion, and second, to the subset of these that reported experiencing *both* an injury and ingestion.

**Table 1:** Frequency of Home Injuries and Ingestions^1^ Since the Start of the U.S. Coronavirus Pandemic and Associated Household Characteristics, Nationally Representative Sample, *N* = 2011, Surveyed June 17–29, 2020, N (Weighted Proportions)

Household Characteristics	Injury and/or Ingestion^**2**^		
	Both	Either	Neither Injury nor Ingestion
**Urbanicity**			
Urban	171 (64)*	254 (45)*	404 (28)
Suburban	80 (30)*	217 (38)*	750 (52)
Rural	18 (7)*	99 (17)	285 (20)
**Census Region**			
Northeast	52 (19)	117 (20)	239 (17)
Midwest	29 (11)*	99 (17)*	327 (23)
South	118 (44)	217 (38)	538 (37)
West	70 (26)	139 (24)	336 (23)
**Combined Pre-Tax Household Income**			
Less than $15,000	19 (7)	36 (6)	110 (8)
$15,000 to $24,999	22 (8)	44 (8)	93 (6)
$25,000 to $34,999	18 (7)	42 (7)	109 (8)
$35,000 to $49,999	23 (9)	44 (8)	177 (12)
$50,000 to $74,999	28 (10)*	84 (15)	265 (18)
$75,000 to $99,999	37 (14)	77 (14)	202 (14)
More than $100,000	122 (45)*	244 (43)*	484 (34)
**Number of Children (< 18 years)**			
0 children	41 (15)*	212 (37)*	1007 (70)
1 child	98 (36)*	158 (28)*	252 (18)
2 children	89 (33)*	133 (23)*	121 (8)
3 children	35 (13)*	54 (9)*	31 (2)
4+ children	6 (2)	14 (2)	28 (2)
**Number of Adults (18+ year old)**^**3**^			
1 adult	73 (27)	127 (22)	308 (21)
2+ adults	196 (73)	444 (78)	1132 (79)
**Changes in Time Spent at Home**			
More time at home	152 (57)*	383 (67)	990 (69)
No change	81 (30)*	123 (22)	282 (20)
Less time at home	32 (12)*	50 (9)*	41 (3)
Varies by person	4 (1)*	15 (3)*	127 (9)

^1^Since the coronavirus pandemic started in the U.S., have you or has anyone living in your home: 1) swallowed or eaten something that could make them sick, like a medication or a household product, a plant, or something else while in or around your home? Please do not include food allergies or food poisoning or poison ivy rashes or a reaction to a medication that was taken correctly; 2) had an injury where any part of their body was hurt while in or around your home? A home injury could be from a fall, a cut or scrape, getting hit by something, or from a burn or from something else that happened in or around your home?

^2^Both injury and ingestion is a subset of Either injury or ingestion

^3^Includes respondent

*Statistically significant in comparison to the Neither group, *p* < 0.05

All proportions presented are weighted to targets from the U.S. Census Bureau’s Current Population Survey for adults. Propensity score weighting adjusted for respondents’ propensity to be online. No estimate of theoretical sampling error was calculated because the online survey is not based on a probability sample. For weighted data, sample sizes within calculations are replaced with effective sample sizes to account for bias that could occur due to weighting. For comparative analyses, statistical comparisons of subgroups were carried out by Quantum using T-tests either for means or for proportions depending on the data, using a 95% confidence level (CL) representing a *p*-value < 0.05.

## Results

Of the 2011 respondents, the mean age was 47.2 years (SD = 17.59). Respondents indicated their: sex as female (51%), male (48%), other (3%); race/ethnicity as white (64%), African American (11%), Hispanic/Latinx (16%), Asian/Pacific Islander (6%), and other (3%). More than one-half (54%) were married or living with a partner; 10% did not complete high school, 56% completed high school, and 34% completed college or more. A majority (56%) were employed; 24% were retired; and the remainder were not employed, were stay-at-home parents or students. The pandemic changed employment for participants, with 24% starting to work at home, 11% becoming unemployed, and 10% being furloughed.

In total, 26% of households reported having experienced an injury, and 15% experienced an ingestion. Twenty-eight percent (28%) of households experienced either, and 13% experienced both. The mean number of injuries and/or ingestions by age of the household members affected were 1.5 for 0–4 year olds, 2.1 for both 5–14 and 15–24 year olds, 2.2 for 25–64 year olds, and 1.1 for those 65 years and older (unweighted *n* = 451). Overall, falls were the most common cause of injury, reported by 32%. Other causes were being cut by something sharp (11%), being cut by running into someone/something (9%), getting bruised by running into something/someone (9%), burns from hot objects/matches (5%), being hit with an object (5%), scald burns (4%), choking (4%), and being bitten by an animal (3%). Ingestions of medications were reported by 6% and household products by 4%.

Table 1 shows that relative to the 72% of households that experienced neither an injury nor an ingestion, those that reported either or both were more likely in urban areas, had household incomes > $100,000, and had children living in them; they were less likely to be in the Midwest and in suburban households. The number of adults living in the home was not associated with experiencing an injury and/or ingestion. Although the majority of respondents reported spending more time at home since the pandemic started, this was not significantly associated with increased likelihood of an injury or ingestion: 69% of the group that experienced neither versus 67% of the group that experienced either and 57% of the group that experienced both reported that the people in their household were spending more time at home. In a separate analysis of households that reported having spent more time at home since the pandemic started, 45% of those with children in the home (unweighted *n* = 497) reported an injury or ingestion and 55% did not, compared to 18% and 82% respectively among households without children (unweighted *n* = 965) (*p* < 0.05, data not shown).

## Conclusions

Among our representative sample, 26% reported having experienced an injury since the pandemic started. By comparison, in 2017, 14.3% of respondents to the NHIS survey reported an injury to someone in their household in the 3 months prior to the survey (Michael E. Martinez, National Center for Health Statistics, personal communication, 9/1/2020). The time periods covered by these two surveys are comparable, given that our survey was fielded (i.e., mid to late June) about 3 months after states started issuing stay at home orders and people began working from home (i.e., mid to late March). Considering ingestions as well as injuries, we found that 28% of households experienced either and 13% experienced both. Falls were the most common cause of injury, which is consistent with other studies of home injuries (Chen et al. 2009; Mack et al. 2013; Gielen et al. 2015; McDonald et al. 2016). Having children living in the home was significantly associated with a higher likelihood of households reporting an injury and/or ingestion, and having more adults in the home was not. Families in urban areas and with higher annual incomes were more likely to report an injury and/or ingestion.

We were surprised that there was no overall association between increased time spent at home and the report of injuries and/or ingestions. A more in-depth understanding of the affected individuals and their activities during the time spent at home is warranted, especially because we found that among the 73% of households that reported spending more time at home, 45% of those with children compared to 18% of those without children in the home reported having experienced an injury or ingestion since the pandemic started. Given that many children are still learning remotely and may be for quite some time, there is an urgent need to promote home safety precautions.

The study was limited in that it did not measure the actual amount of time spent in the home or the duration of the stay-at-home guidelines in the respondent’s area. We also lacked information on the intentionality or severity of the reported injuries/ingestions. Nevertheless, results provide the first evidence to our knowledge that during this pandemic many households have experienced injuries, including ingestions, most of which are likely preventable.

Results demonstrate the value of systematically collecting national data on home injuries using an on-line survey, which could be a cost-efficient methodology to supplement the more extensive and expensive National Health Interview Survey, which last reported on injuries in 2007. The rates we found were for roughly a 3-month period, and because people are still largely staying home to prevent the spread of COVID-19, we can expect these rates to be higher in the future. A near real time home injury surveillance system would be a timely addition to our injury prevention tool kit. Findings from this first survey can be used as a baseline measure as well as to create messaging that promotes the need to stay home to prevent the spread of COVID-19 and the importance of home safety precautions.

## Data Availability

The datasets used and analyzed during the current study are available from the corresponding author on reasonable request.
